# Sleep Quality of Functional Gastrointestinal Disorder Patients in Class-Three Hospitals: A Cross-Sectional Study in Tianjin, China

**DOI:** 10.1155/2018/3619748

**Published:** 2018-06-07

**Authors:** Wei Zhao, Hong Jin, Mengque Xu, Dongxu Wang, Yandi Liu, Yanping Tang, Qiuzan Zhang, Jianping Hua, Bangmao Wang

**Affiliations:** ^1^Department of Digestive Diseases, General Hospital of Tianjin Medical University, Tianjin 300052, China; ^2^Department of Gastroenterology, Sir Run Run Shaw Hospital, Zhejiang University, Hangzhou, Zhejiang 310016, China; ^3^Department of Gastroenterology, The 254 Hospital of the People's Liberation Army, Tianjin 300142, China; ^4^Department of Gastroenterology, Tianjin Union Medical Center, Tianjin 300121, China; ^5^Department of Gastroenterology, Nankai Hospital of Tianjin, Tianjin 300100, China; ^6^Department of Gastroenterology, Tianjin 4th Center Hospital, Tianjin 300140, China; ^7^Department of Gastroenterology, Tianjin First Center Hospital, Tianjin 300192, China

## Abstract

**Background:**

Functional gastrointestinal disorder (FGID) patients are influenced by anxiety, depression, and low sleep quality, which reduce the quality of their life. However, epidemiological data on the quality of sleep in FGID patients were lacking. This study aims to explore the sleep quality and influencing factors of the sleep quality in FGID patients.

**Methods:**

1200 subjects, diagnosed as FGID in one of the six class-three hospitals in Tianjin, China, from January to December 2014, were recruited. The information about demographic information, the severity of clinical symptoms, psychological status (Zung self-rating depression scale), and sleep quality (evaluated with Pittsburgh sleep quality index) was gathered.

**Results:**

The questionnaires from 1117 participants were collected including 920 of functional dyspepsia (FD) patients, 77 of irritable bowel disease (IBS) patients, 26 of functional constipation (FC) patients, and 94 other FGID patients. The results showed that morbidity rate for FD patients who had sleep disorders was higher than those who suffered from IBS or FC (*P* < 0.001). The proportion of elderly patients suffering from low sleep quality was higher than that of middle-aged and young patients (*P* < 0.001). The binary logistic regression analysis showed that age, education, and the severity of FGID symptom were influencing factors for poor sleep quality in FGID patients.

**Conclusion:**

The issue of poor sleep quality in FGID patients in Tianjin area is prominent, and elderly patients suffer lower sleep quality than other FGID patients. Age, education, and the severity of FGID symptoms are critical influencing factors which result in a drop-in sleep quality.

## 1. Introduction

Functional gastrointestinal disorder (FGID) includes some symptoms of morphologic and physiological abnormalities in different parts of the digestive systems. FGID, such as functional dyspepsia (FD), functional constipation (FC), irritable bowel disease (IBS), and other symptoms, lowered the life quality of patients [1]. Patients with FGID have to spend money on medicine and visit hospital more often, which bring substantial economic burden for both patients' families and medical systems [2].

The morbidity of FGID in China ranges from 5.67% to 55.24% [3, 4]. The previous study showed that besides the digestive system the symptoms were associated with issues in psychological status and sleep disorders [5, 6]. Thus, it is crucial to evaluate the correlation between mental status, sleep quality, and severity of FGID symptoms in those individuals [7]. However, there are little data about the relationship between epidemiology of FGID and sleep quality in China, especially the data related to the epidemiological investigation of sleep disorders. Thus, this research attempts to discuss the relationship between sleep quality and mental and psychological conditions in different FGID patients at six class-three hospitals in Tianjin.

## 2. Materials and Methods

The present study was approved by Ethics Committee of the General Hospital of Tianjin Medical University (2013030079). All the enrolled subjects have signed the informed consent. A total of 1200 patients with FGID were recruited in six class-three hospitals (involving patients from all districts in Tianjin) from January to December in 2014. Questionnaires were used to collect health-related information of patients. Examinations of blood, urine, and stool, liver and kidney function, blood pressure, blood sugar, thyroid function, chest radiograph, gastrointestinal endoscopy, ultrasonography, and electrocardiogram were conducted for all the subjects before the investigation. The inclusion criteria of FGID are the FGID Rome III standard, and patients aged from 18 to 65 years old [8]. The exclusion criteria involved extensive digestive system organic disease, malignant tumor, neuropsychiatric disorder, diseases in circulatory, respiratory, metabolic and endocrine system diseases, and patients unable to participate in the investigation.

10 trained investigators conducted the interview in the follow-up clinic of the hospital using structured questionnaires (after and before investigation). Participants answered questionnaires (Supplementary file 1) with the assistance of professionals who have received unified training before the investigation. Information on their demographic data and clinical symptoms and their severity of the symptoms, psychological condition, and sleep quality was all collected. Each questionnaire has been examined by the interviewer after participants completed it, and we adopted parallel double entry system.

Psychological status was evaluated with Zung self-rating depression scale. The patients' psychological status with their actual feelings and judgment within one week were rated. If SAS score is higher than 50, the patients are considered to suffer from anxiety; if SAS is higher than 53, they are considered to be suffering from depression [9, 10]. According to the criteria of age classification by WHO reported in 2012, age range is divided into three groups: young (18–44 years), middle (44–59 years), and old (>60 years) [11].

Assessment of sleep quality was finished with the Pittsburgh sleep quality index (PSQI) to reflect patients' sleep quality over one month through its seven dimensions, namely, subjective sleep quality, sleep latency (i.e., how long it takes to fall asleep), sleep duration, habitual sleep efficiency (i.e., the percentage of time in bed that one is asleep), sleep disturbances, use of sleeping medication, and daytime dysfunction [12]. The maximum score is 21, and if the overall score is greater than 8, the patients are shown to be suffering from a sleep disorder, and the higher the score is, the lower their sleep quality can be [12].

## 3. Statistical Analysis

The database was input by EpiData3.0 and analyzed by SAS 9.3. The data with normal distribution was shown as the mean ± standard deviation, and those with abnormal distribution were displayed as median and range. One-way ANOVA and rank-sum test were used to compare the difference between the groups. Ratio comparison was performed with the chi-square test, and binary logistic regression analysis was used to analyze the risk factors for sleep disorders. *P* < 0.05 was regarded as statistical significance.

## 4. Results

The current investigation recovered 1117 questionnaires, including 466 males and 649 females, with an average age of 50.8 ± 11.7. Among all subjects, there were 920 FD patients, 349 males and 571 females, with an age of 50.9 ± 11.5; 77 IBS patients, 53 males and 24 females, with an age of 49.7 ± 12.1; 24 FC patients, 18 males and 6 females, with an age of 53.0 ± 10.9; 94 other types of FGID patients, 63 males with an age of 49.8 ± 13.3. Overall, there is no significant difference among all groups.

Among all the investigated FGID patients, the incidence of sleep disorder of each group varied within 71.28%–87.10%; the incidence of suffering from both a sleep disorder and abnormal psychological problems was 69.36%–83.56%, while the incidence of no sleep disorder and abnormal psychological problems was only 0–4.26%. The incidence of sleep disorder of FD patients was 87.10%; their incidence of suffering from both sleep disorder and abnormal psychological problems was 83.59%, higher than other types of FGIDs. A substantial difference between the abnormal psychological problem rations did not exist among groups (Table 1).

The enrolled patients were assigned to different groups according to their age. The results demonstrate that there was no statistical difference in the course of disease and gender among the young, middle-aged, and elderly groups, while there was a difference in education (with the young group having a higher ratio of well-educated patients). Age affected the sleep quality of FGID patients, as seen in the results. Compared with the young and the middle-aged, the elderly stood a higher ratio of sleep disorder and together with abnormal psychological problems, a higher overall score for sleep quality. There was no significant difference in the ratio of abnormal psychological problems among all groups, as seen in Table 2.

The study was conducted through binary logistic regression analysis to explore risk factors of sleep disorder in FGIDs patients as they appear at advanced ages, with low levels of education, and severity of symptoms being seen as significant risk factors when it comes to sleep disorder (Table 3).

## 5. Discussion

Sleep disorders severely affect adults' quality of life and contribute to factors leading to the death rate [13, 14]. Thus, it is crucial to investigate this matter in the interests of public health. FGIDs are common clinical diseases often accompanied by psychological and sleep disorders. In recent years, psychological and sleep disorder problems in FGIDs have gradually attracted public attention [15, 16]. Jiang et al. found that not only have the symptoms of gastrointestinal tracts of refractory FD patients become more and more severe, but also their psychological and sleep disorders become more prominent, and these are considered to be the key reasons why symptoms come up again and again [17]. If psychological and sleep problems can be correctly identified and handled promptly, the overall symptoms and quality of life of those FGIDs will be improved. However, current epidemiological investigation into FGIDs still needs further perfection [18].

The present study finds that the ratio of FGIDs (including FD, IBS, and FC) is relatively high (71.77%–87.10%), and compared with other types of FGIDs, FD patients suffer from sleep problems more easily. The result is different from that of Schurman et al. in which 283 subjects with age between 8 and 17, with the Sleep Disturbances Scale for Children (SDSC) used to evaluate sleep disorder and its incidence was shown to be 45%, with no significant difference among all FGIDs [16]. The main reason for this difference may lie in the age range of subjects, number of samples, and different evaluation methods to assess psychological and sleep disorders. With epidemiological studies on a large sample in multicenters (subjects: 3600, age: 18–80), Wu et al. found that 22.16% of FGIDs suffered excessive daytime sleepiness (EDS) (assessed by Epworth sleepiness score), and the situation is the most severe in ulcer-like dyspepsia and gallbladder dysfunction [3]. One potential cause of our results that different from the previously study is the index measuring sleep disorder and demographic characteristics of enrolled patients.

Age affects sleep quality [19] and the situation becomes more prominent when it comes to the elderly with greater harms [20]. The present study shows that the ratio of sleep disorder and its complications with abnormal psychological problems is remarkably higher than the young and the middle-aged and its dimensions are more abnormal than the latter, which shows that the elderly are faced with more severe sleep problems. The present study finds that level of education is one of the risk factors of sleep quality (estimate = −0.421), where the ratio of low levels of education among the elderly patients might explain their worse sleep quality. The current study does not appraise quality of life across all age groups, so it cannot conclude that sleep disorders can lead to lower quality of life. Therefore, it is speculated that sleep disorders might lead to lower quality of life in elderly FGIDs patients [21, 22]. In the future, experimental design will be improved to specify the relationship between sleep disorder and quality of life of FGIDs patients.

Factors influencing sleep disorders are very complex. Besides advanced age, Tang et al. [23] found that sleep disorders of normal people were related to education, female, high education, being single, living in rural areas, smoking, and drinking. The current study showed that age, seriousness of symptoms, and education were the key risk factors. Subject groups might be the main reason for differences between Tang's research and our study, and regional differences of enrolled patients may also affect the results. As there were limitations in experimental design, the present study cannot conclude that sleep disorders have a cause-and-effect relationship with the above risk factors. The main strength of this manuscript that is the first study explores the relationship between sleep quality and mental and psychological conditions in different FGID patients in North China. But the small sample size is the main limitation.

## 6. Conclusion

This study finds that FGID patients are not only confronted with severe sleep disorders but also suffer from abnormal psychological problems, through epidemiological investigation of multicenters in Tianjin; sleep disorders in the elderly are more prominent. Age, education, and severity of symptoms can be the risk factors contributing to sleep disorder and as there are differences among baseline information among all FGIDs, large samples with more rigorous epidemiological investigation are required.

## Figures and Tables

**Table 1:** The proportion of sleep disorders and mental disorders of FGID patients.

Characters		FD	IBS	FC	Other FGIDs	*F*/*x*^2^	*P*
*n*		920	77	26	94	-	-

Age	mean	50.9 ± 11.5	49.7 ± 12.1	53.0 ± 10.9	49.8 ± 13.3	0.784	0.503

Gender	Male	349	53	18	46	41.62	<0.001
	Female	571	24	6	48		

Education level	Illiteracy	7	3	3	5	49.46	<0.001
	Primary school	77	7	8	9		
	Middle school	536	40	12	46		
	College and above	300	17	4	27		

Sleep disorders	yes	801	63	23	67	18.08	<0.001
	no	119	14	3	27		

Psychological problem	Yes no	871 49	74 3	24 2	88 6	0.80	0.85

Sleep disorders with psychological problem	Yes no	769 151	61 16	21 5	65 29	12.52	0.006

No sleep disorders and psychological problem	Yes no	16 904	1 76	0 26	4 90	3.61	0.306

**Table 2:** Effects of age on sleep quality of FGID patients.

	Age			*F*/*x*2	*P *value
	18–40	41–59	≥60		
*n*	209	629	279	-	-
Gender (F/M)	129/80	394/236	167/112	*χ*2 = 0.59	.745
Education level^*∗*^	4/2/50/153	5/51/378/195	6/57/174/42	*χ*2 = 218.787	*P* < .001
Course of disease (month)	6 (8)	6 (8)	6 (8)	*Z* = 0.663	.718
Sleep disorder (%)	76.56% 160/209	84.10% 529/629	94.98% 265/279	*χ*2 = 34.52	*P* < .001
Psychological problem (%)	96.65% 202/209	93.64% 589/629	95.43% 266/279	*χ*2 = 3.17	0.205
Sleep disorders with Psychological problem (%)	74.64% 156/209	81.56% 513/629	88.53% 247/279	*χ*2 = 15.82	<0.001
PSQI score	10.28 ± 3.10	11.40 ± 8.62	12.65 ± 3.59	*F* = 27.10	*P* < .001
Score of sleep quality	1.50 ± 0.91	1.59 ± 0.98	1.82 ± 0.88	*F* = 8.30	*P* < .001
Score of time to fall asleep	1.52 ± 1.08	1.71 ± 1.02	1.90 ± 1.06	*F* = 7.73	*P* < .001
Sleeping time	1.10 ± 0.70	1.29 ± 0.87	1.56 ± 0.95	*F* = 18.35	*P* < .001
Score of sleep efficiency	2.99 ± 0.14	2.99 ± 0.08	2.96 ± 0.33	*F* = 4.65	.010
Score of sleep disorder	1.20 ± 0.47	1.36 ± 0.57	1.40 ± 0.59	*F* = 8.68	*P* < .001
Score of Hypnotic drugs	0 (0)	0 (1)	0 (2)	*Z* = 52.170	*P* < .001
Score of daily functions	2 (2)	2 (2)	2 (2)	*Z* = 6.526	.038

^*∗*^Education level: illiteracy/primary school/middle school/college and above.

**Table 3:** Analysis of risk factors for sleep disorders of FGID patients.

Characters	OR	OR 95% CI		*P*
		Lower	Upper	
Age group				
Young	1			
Old	1.020	1.003	1.038	0.023
Sex				
Female	1			
Male	1.197	0.808	1.773	0.370
Education level				
Low	1			
High	0.653	0.479	0.909	0.012
Course of disease				
Short-term	1			
long-term	0.990	0.974	1.005	0.190
Severity of symptoms				
Slight	1			
Serious	2.861	1.871	4.376	<0.001
Anxious				
Yes	1			
No	0.755	0.409	1.393	0.349
Depressed				
Yes	1			
No	0.859	0.478	1.541	0.609

## Data Availability

All the raw data are available from Dr. Wei Zhao (wzhao02@tmu.edu.cn) upon request.

## Supplementary Materials

Supplementary Materials 10 trained investigators conducted the interview in the follow-up clinic of the hospital using structured questionnaires (after and before investigation). Participants answered questionnaires (Supplementary file 1).
