# Supplementary material for: Sleep Quality of Functional Gastrointestinal Disorder Patients in Class-Three Hospitals: A Cross-Sectional Study in Tianjin, China
Source: Biomed Res Int. 2018 Jun 7;2018:3619748. doi: 10.1155/2018/3619748 (PMC6011102; doi:10.1155/2018/3619748)
Supplement: Supplementary Materials — 10 trained investigators conducted the interview in the follow-up clinic of the hospital using structured questionnaires (after and before investigation). Participants answered questionnaires (Supplementary file 1). [file 3619748.f1.pdf]

Supplementary file 1. The questionnaires used in this study.

General information

Name		Birth place		Body weight kg	
Gender		Tel		Course of disease (month)	
Age		Height cm		Marriage	
Education level	Illiteracy	Primary school	Middle school	College and above	

Symptom of functional gastrointestinal disease

Here are 19 symptoms related to functional gastrointestinal disorders, in order to understand your condition more comprehensively and provide targeted treatment, please indicate the selection of this item with "√" in "□", according to your actual situation in the past six months.

Notes：

Severity:

Mild: Slight symptoms, need attention to feel

Moderate: obvious symptoms, but does not affect working life

Severe: obvious symptoms, affecting working life

Extremely severe: Conscious symptoms are obvious and seriously affect working life

1. Upper abdominal pain   Yes □ No □

Severity in recent three months:

Mild □ Moderate □ Severe□ Extremely severe □

Frequency of Occurrence：

once every week□   2-4 times per week □

5-6 times per week□   happens all the day □

Improved after defecation or exhaust：

Yes □   No □

2. Big belly   Yes □ No □

Severity in recent three months:

Mild □ Moderate □ Severe□ Extremely severe □

Frequency of Occurrence：

once every week□   2-4 times per week □

5-6 times per week□   happens all the day □

3. Full swelling discomfort after meal   Yes □ No □

Severity in recent three months:

Mild □ Moderate □ Severe□ Extremely severe □

Frequency of Occurrence：
once every week□ 2-4 times per week □
5-6 times per week□ happens all the day □
4. Early satiety Yes □ No □
Severity in recent three months:
Mild □ Moderate □ Severe□ Extremely severe □
Frequency of Occurrence：
once every week□ 2-4 times per week □
5-6 times per week□ happens all the day □
5 . Disgusting after meals Yes □ No □
Severity in recent three months:
Mild □ Moderate □ Severe□ Extremely severe □
Frequency of Occurrence：
once every week□ 2-4 times per week □
5-6 times per week□ happens all the day □
6 . Belching Yes □ No □
Severity in recent three months:
Mild □ Moderate □ Severe□ Extremely severe □
Frequency of Occurrence：
once every week□ 2-4 times per week □
5-6 times per week□ happens all the day □
7. Burning sensation in the abdomen Yes □ No □
Severity in recent three months:
Mild □ Moderate □ Severe□ Extremely severe □
Frequency of Occurrence：
once every week□ 2-4 times per week □
5-6 times per week□ happens all the day □
Improved after defecation or exhaust：
Yes □ No □
8. Acid reflux Yes □ No □
Severity in recent three months:
Mild □ Moderate □ Severe□ Extremely severe □
Frequency of Occurrence：
once every week□ 2-4 times per week □
5-6 times per week□ happens all the day □
9. Vomit Yes □ No □
Severity in recent three months:
Mild □ Moderate □ Severe□ Extremely severe □
Frequency of Occurrence：
once every week□ 2-4 times per week □
5-6 times per week□ happens all the day □
10. Sensation or discomfort of foreign body in pharynx Yes □ No □
Severity in recent three months:

Mild □ Moderate □ Severe□ Extremely severe □
Frequency of Occurrence：
once every week□ 2-4 times per week □
5-6 times per week□ happens all the day □
11. Lower abdominal pain Yes □ No □
Severity in recent three months:
Mild □ Moderate □ Severe□ Extremely severe □
Frequency of Occurrence：
once every week□ 2-4 times per week □
5-6 times per week□ happens all the day □
Improved after defecation：
Yes □ No □
12. Lower abdomen discomfort (not painful) Yes □ No □
Severity in recent three months:
Mild □ Moderate □ Severe□ Extremely severe □
Frequency of Occurrence：
once every week□ 2-4 times per week □
5-6 times per week□ happens all the day □
Improved after defecation：
Yes □ No □
Are there the following symptoms of abdominal pain and abdominal discomfort?
Change of defecation frequency Yes □ No □
Change of stool trait (shape) Yes □ No □
13. Sheep dung or hard poop Yes □ No □
Severity in recent three months:
Mild □ Moderate □ Severe□ Extremely severe □
Frequency of Occurrence：
1-2 days per month □ 3-6 days per month □
7-10 days per month □ more than 10 days per month □
14. defecate difficulty Yes □ No □
Severity in recent three months:
Mild □ Moderate □ Severe□ Extremely severe □
Frequency of Occurrence：
1-2 days per month □ 3-6 days per month □
7-10 days per month □ more than 10 days per month □
15. Dilute (soft) or watery Yes □ No □
Severity in recent three months:
Mild □ Moderate □ Severe□ Extremely severe □
Frequency of Occurrence：
1-2 days per month □ 3-6 days per month □
7-10 days per month □ more than 10 days per month □
16. No sense of defecation Yes □ No □
Severity in recent three months:

Mild □ Moderate □ Severe□ Extremely severe □

Frequency of Occurrence：

1-2 days per month □ 3-6 days per month □

7-10 days per month □ more than 10 days per month □

17. anxious defecation Yes □ No □

Severity in recent three months:

Mild □ Moderate □ Severe□ Extremely severe □

Frequency of Occurrence：

1-2 days per month □ 3-6 days per month □

7-10 days per month □ more than 10 days per month □

18. Defecation less than 3 times a week Yes □ No □

Severity in recent three months:

Mild □ Moderate □ Severe□ Extremely severe □

Frequency of Occurrence：

one week per month □ 2 weeks per month □

3 weeks per month □ 4 weeks per month □

19. Defecation more than 3 times per day Yes □ No □

Severity in recent three months:

Mild □ Moderate □ Severe□ Extremely severe □

Frequency of Occurrence：

1-2 days per month □ 3-6 days per month □

7-10 days per month □ more than 10 days per month □

The above 19 symptoms that have plagued you for the last six months (choose only one)：

1 □ 2 □ 3 □ 4 □ 5 □

6 □ 7 □ 8 □ 9 □ 10 □

11 □ 12 □ 13 □ 14 □ 15 □

16 □ 17 □ 18 □ 19 □

Pittsburgh sleep quality index PSQI

Please answer the following questions!

Here are some questions about your sleep in the last 1 months, please select or fill in the answers that best fit your actual situation for the last 1 months.

1. Usually you go to bed at __ o'clock.
2. Usually it takes __ to fall sleep.
3. Usually you get up at __ o'clock.
4. Usually __ hours actual sleep per night (not equal to bed time)
5. Please choose one of the following conditions affect sleep and worry：

a. Difficulty in falling asleep (no sleep in 30 minutes) (1) (2) 〈1 times/week (3)1-2 Times/week (4)≥3 Times/week

b. Early Awakening at night (1) no (2) 〈1 times/week (3)1-2 Times/week (4)≥3 Times/week

c. Go to the toilet at night (1) no (2) 〈1 times/week (3)1-2 Times/week (4)≥3 Times/week

d. Poor breathing (1) no (2) 〈1 times/week (3)1-2 Times/week (4)≥3 Times/week

e. Cough or Snore high (1) no (2) 〈1 times/week (3)1-2 Times/week (4)≥3 Times/week

f. feeling cold (1) no (2) 〈1 times/week (3)1-2 Times/week (4)≥3 Times/week

g. feeling hot (1) no (2) 〈1 times/week (3)1-2 Times/week (4)≥3 Times/week

h. Nightmares (1) no (2) 〈1 times/week (3)1-2 Times/week (4)≥3 Times/week

i. Pain or discomfort (1) no (2) 〈1 times/week (3)1-2 Times/week (4)≥3 Times/week

j. Other things that affect sleep (1) no (2) 〈1 times/week (3)1-2 Times/week (4)≥3 Times/week

If yes, please specify：

6. In general, you think your sleep quality (1) very good (2) better (3) poor (4) very poor
7. You use drug hypnosis (1) no (2) 〈1 times/week (3)1-2 Times/week (4)≥3 Times/week
8. Do you often feel sleepy (1) no (2) 〈1 times/weeks (3)1-2 times/week (4)≥3 Times/week
9. Do you have enough energy to do things (1) not (2) occasionally have (3) sometimes have (4) often have

PHQ-4 Patient Emotion Questionnaire

How long have you been bothered by the following questions over the past 2 weeks?

	Not at all.<br>0 points	Sone days<br>1 points	More than half of the days<br>2 points	Almost every day<br>3 points
To feel nervous, restless, or prone to anger				
Unable to stop or control worry				
Feel depressed and hopeless				
Have no interest or pleasure in doing things				

Zung self-rating depression scale 1

Here are 20 items, please read each one carefully, make it clear, and then write " √ " according to the actual feeling of your week in the appropriate column, please do not miss any of the project, and do not write more than 2 columns in the same line.

	Have no or little time to have	sometim es	most times	Most or all of the time
1. I feel more nervous and anxious than usual				
2. I feel scared for no reason				
3. I am easily upset or alarmed				
4. I think I may be going crazy				
5. I think everything is good, no unfortunate				
6. I tremble hands and feet				
7. I am suffering from headache, neck pain and back pain				
8. I feel vulnerable to weakness and fatigue				
9. I feel calm, and easy to sit quietly				
10. I feel a rapid heartbeat				
11. I am distracted by a dizziness				
12. I have fainted, or feel like collapsed				
13. I feel exhaled easily				
14. My hands and feet numb and tingling				
15. I am distressed because of stomach ache and indigestion				
16. I often have to urinate				
17. My hands and feet are often dry and warm				
18. I'm hot and blushing				
19. I am easy to fall asleep and sleep well overnight				
20. I have a bad dream				

Zung Zung self-rating depression scale 2

Here are 20 items, please read each one carefully, make it clear, and then write " √ " according to the actual feeling of your week in the appropriate column, please do not miss any of the project, and do not write more than 2 columns in the same line.

	Have no or little time to have	sometimes	most times	Most or all of the time
1. I feel depressed, depressed				
2. I feel good in the morning				
3. I want to cry or cry				
4. I do not sleep well at night				
5. I eat as much as usual				
6. My sexual function is normal				
7. I feel weight loss				
8. I'm worried about constipation				
9. My heart beat faster than usual				
10. I feel tired for no reason				
11. My mind is as clear as usual				
12. I do things as usual do not feel difficult				
13. I am restless and hard to keep calm				
14. I am hopeful for the future				
15. I am more easily irritated than usual				
16. I think it's easy to decide what				
17. I feel that I am a useful and indispensable person				
18. My life is meaningful				
19. If I die, others will be fare better				
20. I still like my favorite things				

Summary

Are your symptoms related to sleep?	Yes □ Not sure □ No □<br>If relevant, symptoms cause poor sleep □<br>poor sleep cause symptoms □
How do you treat sleep problems	A. Have you ever seen a psych clinic?<br>Never □<br>Sometimes □<br>Regularly □<br>B. Have you seen the department of Neurology?<br>Never □<br>Sometimes □<br>Regularly □<br><br>C. Use of sleeping pills<br>Never □ Sometimes □<br>quite often □ Almost everyday □<br>What kind of sleeping pill to take： __<br>dosage： __<br>Days taken: __<br><br>D What do you think of sleeping pills?<br>Sleeping pills worked. □<br>Sleeping pills are getting worse. □
